# Why Do Female *Callosobruchus maculatus* Kick Their Mates?

**DOI:** 10.1371/journal.pone.0095747

**Published:** 2014-04-21

**Authors:** Emile van Lieshout, Kathryn B. McNamara, Leigh W. Simmons

**Affiliations:** Centre for Evolutionary Biology, School of Animal Biology (M092), University of Western Australia, Crawley, Australia; University of Melbourne, Australia

## Abstract

Sexual conflict is now recognised as an important driver of sexual trait evolution. However, due to their variable outcomes and effects on other fitness components, the detection of sexual conflicts on individual traits can be complicated. This difficulty is exemplified in the beetle *Callosobruchus maculatus*, where longer matings increase the size of nutritious ejaculates but simultaneously reduce female future receptivity. While previous studies show that females gain direct benefits from extended mating duration, females show conspicuous copulatory kicking behaviour, apparently to dislodge mating males prematurely. We explore the potential for sexual conflict by comparing several fitness components and remating propensity in pairs of full sibling females where each female mated with a male from an unrelated pair of full sibling males. For one female, matings were terminated at the onset of kicking, whereas the other’s matings remained uninterrupted. While fecundity (number of eggs) was similar between treatments, uninterrupted matings enhanced adult offspring numbers and fractionally also longevity. However, females whose matings were interrupted at the onset of kicking exhibited an increased propensity to remate. Since polyandry can benefit female fitness in this species, we argue that kicking, rather than being maladaptive, may indicate that females prefer remating over increased ejaculate size. It may thus be difficult to assess the presence of sexual conflict over contested traits such as mating duration when females face a trade off between direct benefits gained from one mating and indirect benefits from additional matings.

## Introduction

Sexual conflict, the conflict between the evolutionary interests of individuals of the two sexes [1], is a fundamental driver of adaptation. Any trait that reduces genetic fitness in the other sex by definition imposes antagonistic selection on that sex for traits that counteract this cost [2]. Such conflicts can occur over traits like mating duration, where male and female adaptations and counteradaptations vie for sex-specific optima. Discrepancies between the costs of mating and resistance may ‘resolve’ conflicts in one sex’s favour [3], although on a population level the linkage of male and female average fitness in populations at equal sex ratios should cause similar fitness declines in both sexes [4]. In other cases, ongoing coevolutionary processes can ultimately leave contested traits largely unchanged [5], although the suboptimal trait state for both sexes and the costs of engaging in antagonistic interactions similarly reduce fitness [2]. Assessment of sexual conflict may be further complicated when the consequences of sexual interactions manifest indirectly. This can occur when the direct costs of manipulation to female fecundity are outweighed by sexy-son-type benefits [3] or conversely when competitive males sire offspring of low fitness [6]. As a consequence, sexual conflicts may be difficult to detect, whichever way they are resolved.

A prime illustration of the complexity involved in demonstrating sexual conflict can be found in the seed beetle *Callosobruchus maculatus*. In this species, mating duration increases the degree of damage to the female reproductive tract made by male genital spines [7]. Longer matings also result in the transfer of larger ejaculates [8], [9], which confer direct benefits [10] as well as costs [11] and may contain products that suppress remating [12]. Given these findings, the conspicuous kicking behaviour that females display in the last third of the mating [13] is generally interpreted as an attempt to dislodge the male and limit mating duration [14]. Females commence kicking earlier when mating with large males, which transfer ejaculate at a higher rate, suggesting the onset of kicking marks the receipt of a threshold quantity of ejaculate [9]. In turn, the spiny genitalia of males have been suggested to be a counteradaptation to resist dislodgement by females [14], although recent findings suggest that their length is not associated with variation in mating duration [15] and they serve to promote coupling [16] and the transmission of seminal products through the wall of the female reproductive tract [17].

Several studies have tested the idea that *C. maculatus* exhibits sexual conflict over mating duration. While theory suggests that male mating effort should covary positively with female fecundity [18], larger (and therefore more fecund) females are able to reduce mating duration and thus the size of the ejaculate received [9]. However, females evolved in high-sexual-conflict environments do not exhibit reduced mating duration against standard partners, and variation in the onset of kicking does not affect mating duration [9]. When female kicking legs are ablated, matings last longer and result in more damage from the genital spines [7] and females indeed suffer reduced fitness, although apparently without any benefit to males [14]. Edvardsson and Canal [8] examined conflict over mating duration by either terminating matings at the mean onset of kicking (assumed to be the female optimum), preventing females from kicking (the male optimum), or leaving matings unmanipulated (‘contested’). While the onset of kicking could conceivably occur earlier than the female’s optimal mating duration because kicking does not dislodge males immediately, it should approach female optima. Edvardsson and Canal [8] found that females experience increased fecundity after male-optimal matings, and detected no fitness difference between female-optimal and contested durations, suggesting that no sexual conflict exists. In this previous experiment, however, treatments were set to rough estimates of the typical onset of kicking and the durations of unmanipulated and male-controlled matings, ignoring considerable between-individual variation.

Here, we examine the existence of sexual conflict over mating duration by manipulating mating duration and looking at its effect on several fitness components and remating propensity in single- and twice-mated female *C. maculatus*. Following Edvardsson and Canal [8], we assume that the onset of kicking indicates that mating duration is approaching the female optimum. We improve on previous designs by terminating copulations at the exact onset of kicking in some females while allowing copulations to end naturally in their siblings. Given that ejaculates have properties that induce a refractory period [12], we expect remating propensity to increase when matings are interrupted at the onset of kicking. However, the fitness consequences of female mating duration may depend on mating frequency.

## Materials and Methods

Experimental animals were sourced from a large outbred population that originated from a stock culture held by the Stored Grain Research Laboratory of CSIRO (Canberra, Australia). Under Australian guidelines, animal ethics approval for research on this species is not required. Experimental and stock individuals were maintained under constant conditions at the University of Western Australia for approximately 4 years at 30°C under a light cycle of 12 h light∶12 h dark. Individuals were reared from eggs until adult on black-eyed beans (*Vigna unguiculata*) in several large populations of approximately 300 individuals to ensure a large effective population size.

To create individuals with known relationships, 40 virgin stock males were mated monandrously to 40 virgin stock females. Females were placed in 60 ml vials with approximately 40 black-eyed beans and allowed to oviposit. Males were discarded. Beans that contained larvae were isolated in microtubes just prior to adult emergence. As adults emerged they were isolated in separate microtubes and their emergence date, sex and weight were recorded. For each of the 40 parental pairs, 4 male and 4 female offspring were retained. All experimental animals were between one and three days old when assayed.

To examine the effect of female control over mating duration and subsequent reproductive success in once-mated females, two randomly assigned female siblings were mated to two male siblings from another family. The time taken for copulation to commence, the time taken for females to commence kicking, and the time for females to eject the aedeagus were recorded. For one of the sibling females, the male’s abdomen was severed immediately following the commencement of kicking. This causes the male’s aedeagus to deflate, allowing the female to eject the male’s aedeagus more quickly (Kruskal-Wallis χ^2^_1_ = 53.67, P<0.0001). Male genital spines are located on the inflatable sac at the apex of the aedeagus and are exposed only upon inflation [19]. Hence, deflation of the sac also prevents further damage. The other sibling female’s matings were not interrupted to reflect contested mating durations.

To examine the effect of female control over mating on remating propensity and reproductive success in twice-mated females, we used the remaining two siblings. Females were mated as above and then isolated in microtubes. Here, after 24 h, the females were placed with a newly emerged virgin stock male and allowed the opportunity to remate within ten minutes. If remating did occur, females received the same mating treatment as on their first day: for one, the male’s abdomen was severed at the commencement of kicking, whereas for her sister, the copulation was uninterrupted. Again, the time taken until copulation, until the commencement of kicking, and until the aedeagus was ejected from the female were recorded.

To assess fitness consequences, females were placed into individual 60 ml vials containing 9 g of black-eyed beans immediately following the once- and twice-mated treatments and allowed to oviposit until death. The number of eggs visible on the exterior of each bean (fecundity), the number of adult offspring that subsequently emerged from these beans (fertilisation success) and the female’s longevity were recorded.

### Statistics

Data were analysed using generalised linear mixed models, with family as a random factor to account for the sibling design. The significance of the random variable (family) was tested using log-likelihood tests. Data were transformed to normality for analysis, where appropriate. Three trials were excluded from the twice-mated treatment (in one trial, the couple refused to mate, and in the two remaining trials, experimenter error resulted in a failed trial). Dependent variables in linear models were power transformed to maximise normality of residuals. Interactions between mating treatment and frequency were non-significant in all analyses, and were omitted [20].

## Results

### Remating Propensity

**Figure 1:**
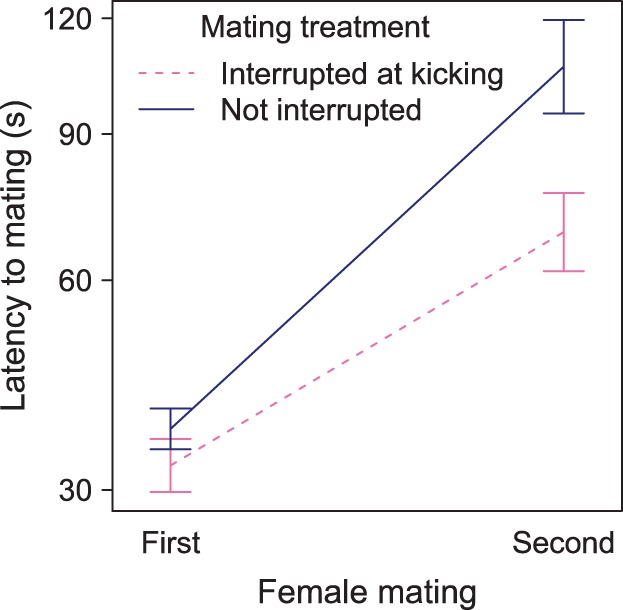
The latency to mating (mean±standard error) increased in the second mating depending on the mating treatment: when a female’s first mating was interrupted at the onset of kicking, the latency to the second mating was reduced (increased remating propensity). Note the exponential scale on the y-axis.

For 75 females that were assigned to mate twice, only 11 failed to re-mate (6 from interrupted and 5 from uninterrupted mating treatments; χ^2^_1_ = 0.14, p = 0.71). To examine the mating treatment on the propensity to remate in the remaining females, we analysed copulation latency using mixed-effects modelling with female and family identity as random factors. Females whose first matings were interrupted at the onset of kicking commenced copulation considerably earlier than females that had received uninterrupted matings (exponent 0.32, mating×treatment, χ^2^_1_ = 4.34, P = 0.037; Fig. 1), but was not affected by the female’s age (χ^2^_1_ = 0.10, P = 0.75), or her weight (χ^2^_1_ = 0.13, P = 0.72). Exclusion of female or family identity did not affect the fit of the model (χ^2^_1_ = 0.27, P = 0.60, χ^2^_1_ = 0.00, P = 1.00 respectively). Furthermore, the median latency to copulation was considerably longer for second matings (32s vs. 72s; χ^2^_1_ = 27.56, P<0.0001).

### Latency to Kicking

The effect of mating treatment on the latency to kicking in the second mating was, like copulation latency, analysed using mixed-effects modelling to account for individual female variation. Kicking latency was not affected by whether the previous copulation duration was interrupted (exponent 0.88, mating×treatment, χ^2^_1_ = 0.26, P = 0.61), nor the female’s age (χ^2^_1_ = 0.02, P = 0.88), or weight (χ^2^_1_ = 1.85, P = 0.17). Exclusion of female or family identity did not affect the fit of the model (χ^2^_1_ = 0.00, P = 0.96, χ^2^_1_ = 0.00, P = 1.00 respectively). Furthermore, the latency to kicking was significantly longer for second matings (χ^2^_1_ = 27.56, P<0.0001).

### Female Reproductive Output

For the 153 females that successfully mated, two single-mated and one double-mated female failed to oviposit. These females were excluded from further analysis. For females that did lay eggs, fecundity increased with female mating frequency (single vs. double medians: 77 vs. 83 eggs; exponent 2.93, χ^2^_1_ = 8.57, P = 0.003), female weight (χ^2^_1_ = 46.14, P<0.0001) and female age at first mating (χ^2^_1_ = 5.45, P = 0.02). Fecundity, however, was not affected by whether the matings were interrupted (χ^2^_1_ = 1.81, P = 0.18). Exclusion of family identity improved the fit of the model (χ^2^_1_ = 5.91, P = 0.02).

**Figure 2:**
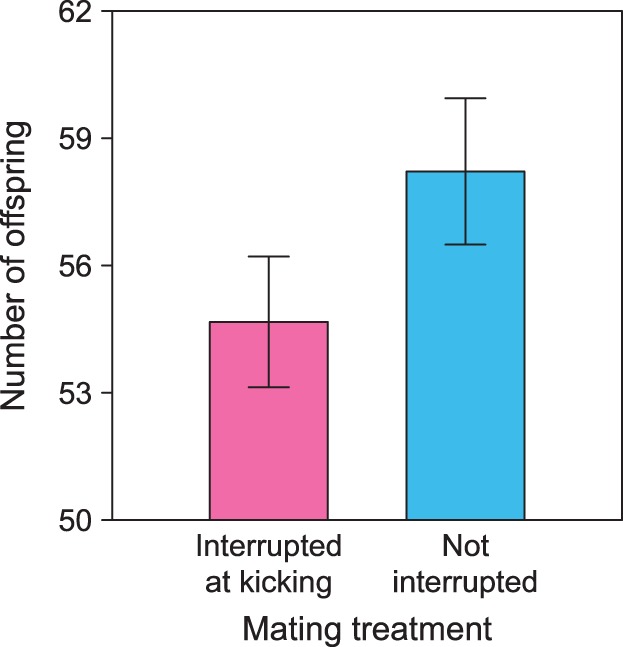
The number of hatched offspring produced by females (mean±standard error) was greater for females when their first mating was uninterrupted rather than terminated at the onset of kicking (interrupted).

One female laid only unviable eggs and was excluded from analysis of offspring production. The number of emerged offspring produced by a female increased when copulation duration was uninterrupted (exponent 2.36, χ^2^_1_ = 7.07, P = 0.008; Fig. 2), and with female weight (χ^2^_1_ = 19.13, P<0.0001). Median offspring numbers in uninterrupted matings were 9% greater than in interrupted matings (61 vs. 56 offspring, respectively). Offspring numbers, however, were not affected by female mating frequency (χ^2^_1_ = 0.53, P = 0.47) or the age of the female at her first mating (χ^2^_1_ = 1.42, P = 0.23). Exclusion of family identity did not affect the fit of the model (χ^2^_1_ = 0.02, P = 0.90).

### Female Longevity

For those females that laid eggs, adult longevity was analysed. Longevity increased subtly but significantly when the matings were uninterrupted (exponent 0.005, χ^2^_1_ = 5.10, P = 0.02; back-transformed means using model exponent: interrupted vs. uninterrupted = 6.98 vs. 7.05 days). Female longevity also increased with the age of the female at her first mating (χ^2^_1_ = 76.72, P<0.0001), and with female weight (χ^2^_1_ = 12.69, P<0.0001). Female longevity, however, was not affected by mating frequency (χ^2^_1_ = 0.88, P = 0.35). Family identity improved the fit of the model (χ^2^_1_ = 4.94, P = 0.03).

## Discussion

In this study we examined whether sexual conflict over mating duration exists in *C. maculatus* by exploring the fitness consequences of interrupting copulations at the onset of female kicking, assuming this indicates that females are approaching their optimal mating duration. We show that some female fitness components clearly benefit from longer copulations. While lifetime fecundity was unaffected by mating duration, both in singly and doubly mated females, uninterrupted copulations slightly increased longevity and resulted in 9% greater offspring numbers. This increase in offspring production is unlikely to be a consequence of sperm limitation in matings interrupted at the onset of kicking: sperm transfer occurs from the start of copulation, and sperm numbers far exceed the requirements for fertilisation [21]. This effect thus appears driven by ejaculate properties associated with mating duration, potentially nutritional content. When mating was terminated at the onset of kicking, however, females had a greater propensity to remate.

Previous work suggests that no conflict over mating duration exists in *C. maculatus* [8]. Indeed, our results agree that receiving longer matings, and the larger ejaculates that accompany these [9], benefit female fitness when compared to interrupted matings at the same mating frequency. Yet, this would suggest that female copulatory kicking behaviour, widespread in seed beetles, is maladaptive yet evolutionarily persistent.

Strictly speaking, the fact that long copulations enhance female fitness does not represent evidence for the absence of conflict over mating duration. For males, the benefits of extending mating duration, increased fecundity, fertility, and paternity share, are obvious [this study; 14]. Yet, for females, both larger ejaculates and multiple mating, especially polyandrously, benefit fitness [22]–[25], in part because both tactics provide direct benefits via ejaculatory water [10]. Our results indicate that, in *C. maculatus*, females clearly show adaptive plasticity in accepting male courtship: when first matings approached female duration optima, females showed a reduced resistance to remating. Although we could not detect an effect on the acceptance of second matings, the cumulative effect of female-controlled mating durations is likely to affect lifetime remating rates. This notion is supported by both by the strong link between mating duration and ejaculate size [8], [9], and the fact that the receptivity-suppressing properties of ejaculates are dose dependent [21], [24].

Relationships between ejaculate size and remating propensity have been reported in other species. In the almond moth *Cadra cautella*, for example, males transfer large spermatophores that inhibit female remating [26]. Females possess chitinous teeth in their reproductive tract, thought to be counteradaptations intended to break down spermatophores and promote female remating [27], [28]. As in the Lepidoptera, females in *C. maculatus* possess teeth in their bursa [29], which may serve a similar function. Inhibition of female remating appears to result from products of the male seminal vesicle [12], although this does not rule out physical effects of ejaculate size. Additionally, there is evidence that female reproductive tract scarring increases with mating duration [7]. Consequently, the apparently premature copulatory kicking of females may indicate that they prefer to gain fitness benefits through increased remating rather than increased ejaculate size. Since remating is unlikely to occur with the same male [22], remating may provide indirect, genetic benefits in addition to direct benefits. Previous studies have shown benefits of polyandry over monandrous multiple mating [22], and fertilization success is in part mediated by the compatibility of male and female genotypes [30]. Yet, females that received uninterrupted matings in our experiment had higher fitness than those whose matings were interrupted. This is perhaps unsurprising, because our maximum mating rate (2) is less than the typical female lifetime mating rate in this population [8], which itself is based on uninterrupted matings.

Male postcopulatory adaptations to reduce sperm competition often directly target female mating duration and frequency [31], to which females may develop a range of behavioural counter-adaptations. For example, a significant proportion of female hide beetles (*Dermestes maculatus*) are able to dislodge mating and mate-guarding males to gain benefits associated with polyandry [32]. Despite the indications of female preferences for shorter matings and increased remating in *C. maculatus*, it is unclear to what extent these are realised in a natural context. Although multiple studies show that ablation of females’ kicking legs results in longer matings [7], [8], van Lieshout, McNamara and Simmons [9] recently showed that the onset of kicking has no effect on the eventual mating duration. However, the evolutionary maintenance of kicking suggests that uninterrupted matings do not fully conform to male optima, and that this conflict is resolved at a suboptimal state for both sexes [2]. Consistent with this, Brown *et al* [33] showed both male and female genetic effects on virgin mating duration.

In conclusion, consistent with other studies, we show that some female fitness components benefit from longer, uninterrupted mating durations. However, we argue that this result alone cannot reveal whether sexual conflict over mating duration exists. By enforcing mating durations to be closer to the female optima, we find indications that the direct fitness benefits of mating duration may trade off with (genetic) benefits gained through additional polyandrous mating. Omission of alternative routes to fitness, such as polyandrous remating, from consideration when testing for sexual conflict could lead to underestimates of its pervasiveness.

## References

[1] Parker GA (1979) Sexual selection and sexual conflict. In: M. S Blum and N. A Blum, editors. Sexual selection and reproductive competition in insects. New York: Academic Press. 123–166.

[2] Lessells CM (2006) The evolutionary outcome of sexual conflict. Philos Trans R Soc Lond B Biol Sci 361: 301–317 10.1098/rstb.2005.1795 16612889 PMC1569608

[3] Kokko H, Brooks R, Jennions MD, Morley J (2003) The evolution of mate choice and mating biases. Proc R Soc Lond B Biol Sci 270: 653–664 10.1098/rspb.2002.2235 PMC1691281 12769467

[4] Arnqvist G, Rowe L (2005) Sexual conflict. Princeton: Princeton University Press. 330 p.

[5] Arnqvist G, Rowe L (2002) Antagonistic coevolution between the sexes in a group of insects. Nature 415: 787–789 10.1038/415787a 11845208

[6] Bilde T, Foged A, Schilling N, Arnqvist G (2009) Postmating sexual selection favors males shat sire offspring with low fitness. Science 324: 1705–1706 10.1126/science.1171675 19556506

[7] Crudgington HS, Siva-Jothy MT (2000) Genital damage, kicking and early death - the battle of the sexes takes a sinister turn in the bean weevil. Nature 407: 855–856 10.1038/35038154 11057654

[8] Edvardsson M, Canal D (2006) The effects of copulation duration in the bruchid beetle *Callosobruchus maculatus* . Behav Ecol 17: 430–434 10.1093/beheco/arj045

[9] van Lieshout E, McNamara KB, Simmons LW (2014) Rapid loss of behavioural plasticity and immunocompetence under intense sexual selection. Evolution accepted. 10.1111/evo.12422 24724572

[10] Edvardsson M (2007) Female *Callosobruchus maculatus* mate when they are thirsty: resource-rich ejaculates as mating effort in a beetle. Anim Behav 74: 183–188 10.1016/j.anbehav.2006.07.018

[11] Eady PE, Hamilton L, Lyons RE (2007) Copulation, genital damage and early death in *Callosobruchus maculatus* . Proc R Soc Lond B Biol Sci 274: 247–252 10.1098/rspb.2006.3710 PMC1685841 17035168

[12] Yamane T, Miyatake T, Kimura Y (2008) Female mating receptivity after injection of male-derived extracts in *Callosobruchus maculatus* . J Insect Physiol 54: 1522–1527 10.1016/j.jinsphys.2008.09.001 18831977

[13] Eady PE (1991) Sperm competition in *Callosobruchus maculatus* (Coleoptera: Bruchidae): a comparison of two methods used to estimate paternity. Ecol Entomol 16: 45–53 10.1111/j.13652311.1991.tb00191.x

[14] Edvardsson M, Tregenza T (2005) Why do male *Callosobruchus maculatus* harm their mates? Behav Ecol 16: 788–793 10.1093/beheco/ari055

[15] Rönn JL, Hotzy C (2012) Do longer genital spines in male seed beetles function as better anchors during mating? Anim Behav 83: 75–79 10.1016/j.anbehav.2011.10.007

[16] Polak M, Rashed A (2010) Microscale laser surgery reveals adaptive function of male intromittent genitalia. Proc R Soc Lond B Biol Sci 277: 1371–1376 10.1098/rspb.2009.1720 PMC2871932 20053645

[17] Hotzy C, Polak M, Rönn JL, Arnqvist G (2012) Phenotypic engineering unveils the function of genital morphology. Curr Biol 22: 2258–2261 10.1016/J.Cub.2012.10.009 23103188

[18] Parker GA, Pizzari T (2010) Sperm competition and ejaculate economics. Biological Reviews 85: 897–934 10.1111/j.1469-185X.2010.00140.x 20560928

[19] Hotzy C, Arnqvist G (2009) Sperm competition favors harmful males in seed beetles. Curr Biol 19: 404–407 10.1016/j.cub.2009.01.045 19230665

[20] Engqvist L (2005) The mistreatment of covariate interaction terms in linear model analyses of behavioural and evolutionary ecology studies. Anim Behav 70: 967–971 10.1016/j.anbehav.2005.01.016

[21] Eady PE (1995) Why do male *Callosobruchus maculatus* beetles inseminate so many sperm? Behav Ecol Sociobiol 36: 25–32 10.1007/BF00175725

[22] Eady PE, Wilson N, Jackson M (2000) Copulating with multiple mates enhances female fecundity but not egg-to-adult survival in the bruchid beetle *Callosobruchus maculatus* . Evolution 54: 2161–2165 10.1111/j.0014-3820.2000.tb01259.x 11209792

[23] Wilson N, Tufton TJ, Eady PE (1999) The effect of single, double, and triple matings on the lifetime fecundity of *Callosobruchus analis* and *Callosobruchus maculatus* (Coleoptera: Bruchidae). J Insect Behav 12: 295–306 10.1023/A:1020883220643

[24] Savalli UM, Fox CW (1999) The effect of male mating history on paternal investment, fecundity and female remating in the seed beetle *Callosobruchus maculatus* . Funct Ecol 13: 167–177 10.1046/j.1365-2435.1999.00287.x

[25] Fox CW (1993) Multiple mating, lifetime fecundity and female mortality of the bruchid beetle, *Callosobruchus maculatus* (Coleoptera: Bruchidae). Funct Ecol 7: 203–208.

[26] McNamara KB, Elgar MA, Jones TM (2008) Seminal compounds, female receptivity and fitness in the almond moth, *Cadra cautella* . Anim Behav 76: 771–777 10.1016/j.anbehav.2008.04.018

[27] Cordero C (2005) The evolutionary origin of signa in female Lepidoptera: natural and sexual selection hypotheses. J Theor Biol 232: 443–449 10.1016/j.jtbi.2004.08.031 15572067

[28] Galicia I, Sánchez V, Cordero C (2008) On the function of signa, a genital trait of female Lepidoptera. Ann Entomol Soc Am 101: 786–793 10.1603/0013-8746(2008)101(786:OTFOSA)2.0.CO;2

[29] Cayetano L, Maklakov AA, Brooks RC, Bonduriansky R (2011) Evolution of male and female genitalia following release from sexual selection. Evolution 65: 2171–2183 10.1111/j.1558-5646.2011.01309.x 21790567

[30] Wilson N, Tubman SC, Eady PE, Robertson GW (1997) Female genotype affects male success in sperm competition. Proc R Soc Lond B Biol Sci 264: 1491–1495 10.1098/rspb.1997.0206

[31] Simmons LW (2001) Sperm competition and its evolutionary consequences in the insects. Princeton, New Jersey: Princeton University Press.

[32] Archer MS, Elgar MA (1999) Female preference for multiple partners: sperm competition in the hide beetle, *Dermestes maculatus* (DeGeer). Anim Behav 58: 669–675 10.1006/anbe.1999.1172 10479383

[33] Brown EA, Gay L, Vasudev R, Tregenza T, Eady PE, et al (2009) Negative phenotypic and genetic associations between copulation duration and longevity in male seed beetles. Heredity 103: 340–345 10.1038/hdy.2009.80 19639006

